# Myc overexpression enhances of epicardial contribution to the developing heart and promotes extensive expansion of the cardiomyocyte population

**DOI:** 10.1038/srep35366

**Published:** 2016-10-18

**Authors:** Cristina Villa del Campo, Ghislaine Lioux, Rita Carmona, Rocío Sierra, Ramón Muñoz-Chápuli, Cristina Clavería, Miguel Torres

**Affiliations:** 1 Centro Nacional de Investigaciones Cardiovasculares (CNIC), c/Melchor Fernández Almagro, 3, E-28029 Madrid, Spain; 2 Department of Animal Biology, Faculty of Science, Campus de Teatinos, University of Málaga, Málaga, Spain; 3 Andalusian Center for Nanomedicine and Biotechnology (BIONAND), c/Severo Ochoa n°25, 29590 Campanillas (Málaga), Spain

## Abstract

Myc is an essential regulator of cell growth and proliferation. Myc overexpression promotes the homeostatic expansion of cardiomyocyte populations by cell competition, however whether this applies to other cardiac lineages remains unknown. The epicardium contributes signals and cells to the developing and adult injured heart and exploring strategies for modulating its activity is of great interest. Using inducible genetic mosaics, we overexpressed Myc in the epicardium and determined the differential expansion of Myc-overexpressing cells with respect to their wild type counterparts. Myc-overexpressing cells overcolonized all epicardial-derived lineages and showed increased ability to invade the myocardium and populate the vasculature. We also found massive colonization of the myocardium by *Wt1Cre*-derived Myc-overexpressing cells, with preservation of cardiac development. Detailed analyses showed that this contribution is unlikely to derive from Cre activity in early cardiomyocytes but does not either derive from established epicardial cells, suggesting that early precursors expressing *Wt1Cre* originate the recombined cardiomyocytes. Myc overexpression does not modify the initial distribution of *Wt1Cre*-recombined cardiomyocytes, indicating that it does not stimulate the incorporation of early expressing *Wt1Cre* lineages to the myocardium, but differentially expands this initial population. We propose that strategies using epicardial lineages for heart repair may benefit from promoting cell competitive ability.

The epicardium is the mesothelium-derived outer layer of the heart. Epicardial cells derive from the proepicardium -a group of cells that bulges from the dorsal pericardial wall-, and are transferred to the heart surface at midgestation (E9.5–10.5 in the mouse)^1^,^2^. Initially epicardial cells conform a squamous single-layered epithelium completely covering the cardiac surface of the heart. From E11, epicardial cells undergo epithelial-mesenchymal transition (EMT) invading and colonizing the subepicardial space and the myocardium^3^,^4^,^5^. Epicardial-derived cells (EPDCs) contribute extensively to the myocardial connective tissues, extensively to smooth muscle and mesenchyme of the coronary vasculature and less so to the endothelium^6^,^7^,^8^,^9^,^10^. In the mouse, but not in other vertebrates, the epicardium has also been reported to contribute to the cardiomyocyte lineage, however controversy remains as to whether these findings derive from undesired recombination of the epicardial Cre lines or represent true contributions^8^,^10^,^11^,^12^.

The Wilms’ tumor gene Wt1 is dynamically expressed in the coelomic epithelium as well as in coelomic epithelium-derived cells in many organs, including the epicardium, therefore several studies have used Wt1 as a lineage marker and tracer for the coelomic and coelomic-derived cells 10,13,14,15,16,17,18. Wt1 expression has also been reported in adult^19^ and embryonic endothelial and endocardial cells 12,20. Wt1 codes for a zinc-finger transcription factor which has been involved in many normal and pathological processes^21^,^22^. The postnatal epicardium is normally quiescent, however it shows cellular and signaling activation upon injury in the fish and the mouse, contributing cells and signals that could be relevant in cardiac repair processes^9^,^23^,^24^,^25^.

Cell competition is a tissue homeostasis mechanism by which low-anabolizing -but otherwise viable- cells are eliminated from tissues due to confrontation with higher-anabolizing cells^26^,^27^,^28^. Increasing anabolism by moderate Myc overexpression in a mosaic fashion leads to cardiomyocyte competition during cardiac development and adult myocardium homeostasis^29^. Cell competition leads to the homeostatic replacement of wild type cardiomyocytes by the Myc-enhanced cardiomyocytes without producing any cardiac anatomical or functional alteration^29^.

Here we studied whether cell competition modifies the myocardial colonization pattern of EPDCs, determining the preferential expansion of Myc-enhanced epicardial cells in the niches usually colonized by EPDCs and in the cardiomyocyte lineage.

## Results

### Myc-overexpression in the WT1-Cre lineage promotes the extensive colonization of the myocardium during cardiac development

**Figure 1:**
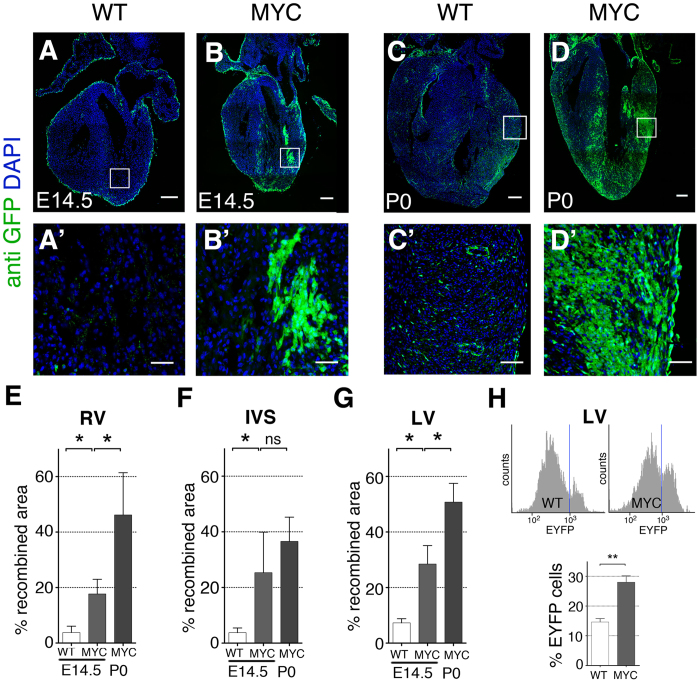
Enhanced myocardial contribution by Myc-overexpressing cells. (**A**–**B**’) Confocal images from histological sections of *iMOS*^*WT*^(WT) (**A**) and *iMOS*^*T1-Myc*^(MYC) (**B**) hearts at E14.5 induced with *Wt1-Cre*. (**A’**,**B’**) show magnification of the boxed areas shown in (**A**,**B**). (**C–D**’) Confocal images from histological sections of P0 *iMOS*^*WT*^(WT) (**C**) and *iMOS*^*T1-Myc*^(MYC) (**D**) recombined with *Wt1-Cre*. (**C’**,**D’**) show magnification of the boxed areas shown in (**C**,**D**). (**E–G**) Quantification of the percentage of recombined area detected in *iMOS*^*WT*^(WT) and *iMOS*^*T1-Myc*^(MYC) at E14.5 and P0 in the RV (**E**), IVS (**F**) and LV (**G**). (**H**) Cytometer histogram plot showing Counts (X axis) versus FITC laser (EYFP detection) in *iMOS*^*WT*^(WT) and *iMOS*^*T1-Myc*^(MYC) E14.5 whole digested hearts (upper panel). Lower panel shows quantification on EYFP population related to the total amount of live cells detected on the cytometer for *iMOS*^*WT*^(WT) and *iMOS*^*T1-Myc*^(MYC) E14.5 hearts. Graphs in E, F, G, H show means ± SEM. *p < 0.05; **p < 0.01; ***p < 0.001. Bar, 200 μm in A, B, C, D and 50 in A’, B’, C’, D’ n ≥ 5 hearts for (**A–G)** and n ≥ 7 for (**H**).

To study whether increased Myc levels modify the behavior and contribution of the epicardial cell lineages, we used the *Wt1Cre* driver^30^ to induce recombination of the *iMOS*^*T1-Myc*^ and *iMOS*^*WT*^ alleles. The iMOS alleles produce an initial 3:1 EYFP:ECFP mosaic^28^. The *iMOS*^*WT*^ allele only expresses the fluorescent reporters and is used as control. The *iMOS*^*T1-Myc*^ allele is similar but overexpresses Myc in the EYFP cells. We analyzed the contribution of the EYFP population to the myocardium in E14.5 hearts. In histological sections, we found that EYFP-Myc cells in *iMOS*^*T1-Myc*^ hearts colonized a larger area than that colonized by their EYFP-wild type equivalent population in the *iMOS*^*WT*^ mice (Fig. 1A–B’, E–G). These results were confirmed by cytometry (Fig. 1H). The greater contribution of the EYFP-Myc population was exacerbated at P0 (Fig. 1C–D’), indicating that the EYFP-Myc cells continued their differential expansion during all cardiac prenatal development. The overcolonization by Myc-overexpressing cells did not induce any change in heart morphology or embryonic development in general.

**Figure 2:**
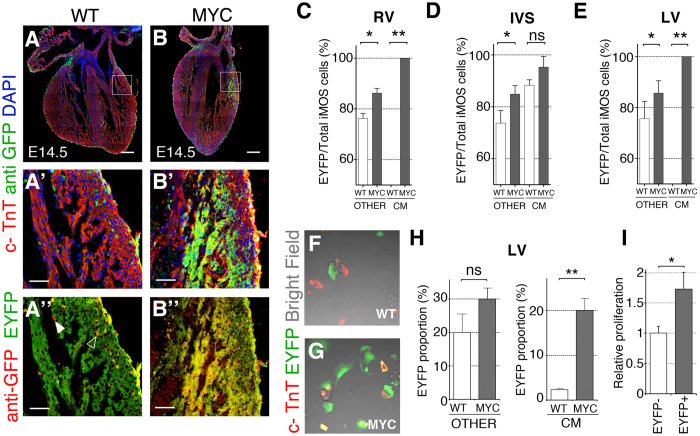
Preferential colonization of the cardiomyocyte lineage by Myc-overexpressing cells. (**A–B**’) Confocal images from histological sections of *iMOS*^*WT*^ (WT) (**A**) and *iMOS*^*T1-Myc*^(MYC) (**B**) hearts at E14.5 induced with *Wt1-Cre*. (**A’**,**B’**) show amplified regions from boxed areas in (**A,B**). (**A”**,**B”**) depict amplified regions from boxed areas in (**A,B**) showing native EYFP and anti-GFP staining. A”, solid indicates an EYFP-negative, anti-GFP-positive cell and empty arrowhead indicates a double-positive cell. (**C–E**) Percentage of EYFP cells within the iMOS-positive population observed at E14.5 in whole hearts of the *iMOS*^*WT*^ (WT) and *iMOS*^*T1-Myc*^(MYC) mosaics in the non-cardiomyocyte fraction and in the cardiomyocyte fraction in the RV (**C**), IVS (**D**) and LV (**E**–**G)** Confocal images showing cultured cells from dissociated E14.5 hearts from *iMOS*^*WT*^(WT) (**F**) and *iMOS*^*T1-Myc*^(MYC) embryos (**G**,**H)** Quantification of the proportion of EYFP cells within the non-cardiomyocyte fraction (left graph) and cardiomyocyte fraction (right graph) from dissociated E14.5 hearts from *iMOS*^*WT*^(WT) and *iMOS*^*T1-Myc*^(MYC) embryos. (**I)**, Quantification of proliferation in cardiomyocytes of the *WT1Cre* lineage. Graph shows the frequency per area unit of Ph3^+^ cardiomyocytes in the EYFP^-^ and EYFP^+^ populations of *Wt1Cre;iMOS*^*T1-Myc*^newborn hearts. Frequencies were normalized to that observed in the EYFP^-^ cardiomyocyte population. Graphs in (**C**–**E**,**H**,**I**) show means +SEM. *p < 0.05; **p < 0.01; ***p < 0.001. Bar, 200 μm in **A**, **B**, and 50 in (**A’**,**B’**,**A”**,**B”**) n ≥ 5 hearts.

We noticed that the expansion of the EYFP-Myc population involved the appearance of large patches of EYFP cardiomyocytes that were not observed in the control mosaics (Fig. 1A’–D’). To further characterize this phenomenon we determined the differential enrichment of the EYFP population within the *Wt1Cre*-recombined ventricular cardiomyocyte compartment and in the non-cardiomyocyte compartment. We found that the EYFP population was moderately expanded in the non-cardiomyocyte compartment of E14.5 *iMOS*^*T1-Myc*^ hearts in comparison with *iMOS*^*WT*^ hearts (Fig. 2A–B”, C–E). In the cardiomyocyte compartment, we observed a non-significant expansion of the EYFP-Myc population over that observed in the control mosaics in the inter-ventricular septum (Fig. 2D). The IVS was the only region in which we could detect *Wt1Cre*-recombined cardiomyocytes in control mosaics (Not shown). In *iMOS*^*T1-Myc*^ mosaics, however, we found extensive presence of iMOS^+^ cardiomyocytes in the RV and LV free walls, which were exclusively EYFP (Fig. 2C,E). These results suggested that most of the observed over-colonization of the ventricular myocardium is due to the appearance of EYFP cardiomyocytes in the free walls of the ventricles. This population is strictly dependent on the presence of Myc overexpression, as it was not observed in the control mosaic and did not include the ECFP population in the *iMOS*^*T1-Myc*^ hearts. To determine the relevance of differential proliferation in the colonization of the ventricles by EYFP-Myc cardiomyocytes, we analyzed the frequency of Ph3^+^ cardiomyocytes in the EYFP^+^ and EYFP^-^ populations of the *Wt1Cre*-recombined *iMOS*^*T1-Myc*^ mosaics. We detected higher proliferative activity of the EYFP-MYC cardiomyocytes, in agreement with the observed over-colonization of the myocardium by this population (Fig. 2I).

### Myc-overexpression in the WT1-Cre lineage increases epicardial cell migration and invasiveness, and promotes the progressive colonization of EPDC compartments

We then studied whether the expansion of EYFP-Myc cells resulted in an overall increase in the number of EPDCs. For this, we established primary cultures from the LV at E14.5 and determined EYFP frequency in the cardiomyocyte and non-cardiomyocyte compartments (Fig. 2F,G). We found a moderate and non-significant increase of the overall abundance of EYFP cells within the non-cardiomyocyte compartment (Fig. 2H). In contrast, the cardiomyocyte compartment showed a ~10-fold increase in EYFP cell frequency (Fig. 2H). These results show that while EYFP-Myc cells expand at the expense of ECFP cells within the EPDC compartment, they do not increase the overall contribution of EPDCs to the myocardium. In contrast, Myc overexpression modifies the contribution of the Wt1Cre lineage in the ventricles to extensively colonize the cardiomyocyte compartment.

**Figure 3:**
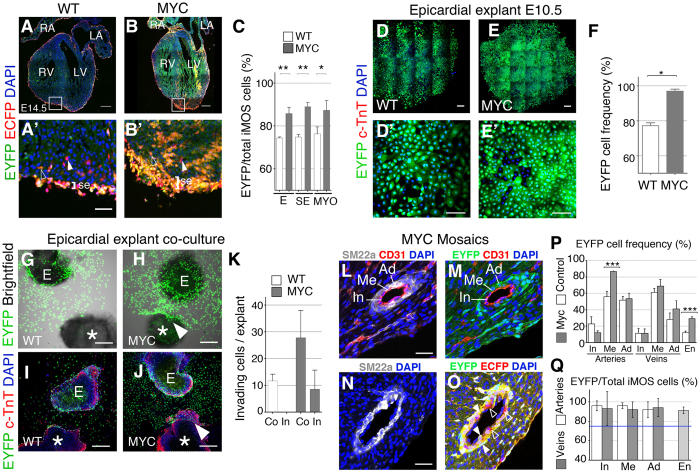
Progressive overcolonization of the epicardium and epicardial derivatives by Myc-overexpressing cells. (**A–B**’) Confocal images from histological sections of *iMOS*^*WT*^(WT) (**A**) and *iMOS*^*T1-Myc*^(MYC) (**B**) hearts at E14.5 induced with *Wt1-Cre*. (**A’**,**B’**) show magnification of the boxed areas in (**A**) and (**B)**. Co-localization of EYFP native fluorescence and anti-GFP immunofluorescence (in red) detects both ECFP (filled arrowheads) and EYFP (open arrowheads) cells. (**C)** frequency of EYFP cells with respect to total iMOS-recombined cells in epicardium (E), subepicardium (SE) and myocardium (MYO). Throughout the figure WT refers to *iMOS*^*WT*^mosaics and MYC to *iMOS*^*T1-Myc*^mosaics. (**D,E**’) 6-day old epicardial cultures and magnifications (**D’**,**E’**). (**F**) Quantification of the EYFP frequency within the epicardial explants. (**G,H)** Co-culture of E10.5 epicardium and a WT E9.0 heart. (**I**,**J)**, Similar cultures as shown in (**G,H)** showing a maximum fluorescence intensity projection (Z = 36 μm). (*) indicates the WT heart and (E) the epicardial explant. Arrowhead shows invading EYFP-Myc epicardial-derived cells. **K** Quantification of epicardial cells per explant that colonize (Co) or invade (In) the WT myocardium. (**L–O)** Confocal image of histological sections from a P0 *iMOS*^*T1-Myc*^heart showing EYFP-Myc perivascular colonization. (**L**–**O**) show the co-localization of vessel layer markers with alone or combined with EYFP and/or anti-GFP (red). EYFP^+^ cells, filled arrowheads, ECFP^+^ cells, open arrowheads. **P** and **Q** show the quantification of EYFP^+^ cell contributions as described in **N**,**O**. The graph above shows the absolute contribution of EYFP cells to the large vessel layers (In: intima, Me: media, Ad: adventitia) and endothelium of the microvasculature (En). **Q** shows the relative contribution of EYFP cells to the total *iMOS*^*+*^ population in *iMOS*^*T1-Myc*^mosaics for the same cell types as in **P**. The blue line in **Q** shows the 75% contribution expected in case of neutral expansion of both populations. Graphs in **C**,**F**,**K**,**P** show means ± SEM. *p < 0.05; **p < 0.01; ***p < 0.001. Graph in **Q** shows means ±95% confidence interval. Bar, 200 μm in **A**,**B**,**D**,**E**, **G**–**J** and 50 μm in **A’**,**B’**,**D’**,**E’**,**L**,**N**. n ≥ 3 hearts in **A**–**C**, n ≥ 5 in **D**–**F**, n ≥ 3 in **G**–**K and** n = 5 in **L**–**P**.

To determine the sequence of enrichment in Myc cells during the development of the epicardial lineage, we analyzed the contribution of the EYFP compartment to the total iMOS^+^ population in the different regions colonized by this lineage. We found that the epicardium, the subepicardial space and the myocardium were all enriched in EYFP-Myc cells (Fig. 3A–C). To confirm these results we studied primary epicardial cultures from E10.5 ventricle explants and found that the EYFP compartment colonized close to 100% of these cultures (Fig. 3D–F). These results indicate the ability of Myc to expand epicardial cells at the expense of wild type cells both *in vitro* and *in vivo* from the earliest stages of epicardial development. We also observed that *in vivo* the enrichment in the sub-epicardial space was increased with respect to that observed in the epicardium, suggesting a higher invasive capacity of Myc-overexpressing cells. To investigate this behavior, we established a co-culture assay, exposing an explanted epicardium-less E9 WT ventricle to an E10.5 iMOS ventricle covered by epicardium (Fig. 3G–J). In these assays, the epicardial cells migrated out from the “donor” ventricle and eventually reached the “receptor” ventricle (Fig. 3G–J). We performed these experiments using either *Wt1Cre*-recombined *iMOS*^*T1-Myc*^ or *iMOS*^*WT*^ donors and observed higher migratory activity of the EYFP-Myc cells and a capacity to invade the receptor myocardium that was not observed for the EYFP-wild type cells (Fig. 3G–K).

We finally studied the contribution of the EYFP cells to the coronary vessels in the postnatal heart (P0) (Fig. 3L–O). At this stage we found variable contribution of *iMOS*^*WT*^*-EYFP*^*+*^ cells to the vessel layers, with limited contribution to the intima (endothelium), strong contribution to the media (smooth muscle) and intermediate to the adventitia (fibroblasts) (Fig. 3L,M,P). The contribution was within a similar range for arteries and veins (Fig. 3P). We then examined the contribution of *EYFP*^*+*^ in *iMOS*^*T1-Myc*^ mosaics (Fig. 3P) and found no increase in the contribution to the intima, non-significant increase for the adventitia and venous media; however, we found a very significant increase in the contribution to the arterial media. Similarly, we found a very important enrichment in EYFP-MYC endothelial cells in the microvasculature (Fig. 3P). We then studied whether the EYFP-Myc population had displaced the EYFP-CFP population in the WT1Cre-derived large vessel cell populations and endothelium of the microvasculature. We determined the enrichment in EYFP^+^ cells within the *iMOS*^*T1-Myc*^-derived cells (Fig. 3N,O) and found that the enrichment in all large vessel layers and endothelium of the microvasculature was close to 100% (Fig. 3Q) and clearly above that observed for the epicardium and subepicardium (Fig. 3C). These results indicate that Myc promotes the progressive expansion of the epicardial lineages starting at the colonization of heart surface and continuously during the colonization of the myocardium and large vessels. This overcolonization takes place mostly at the expense of the WT epicardial-derived compartment, as only mild expansion of the overall *WT1Cre* lineage was observed for the media and the intima, with the notable exception of the arterial smooth muscle layer and the endothelium of the microvasculature, in which Myc cells expanded further and replaced the non-EPDC compartment.

### *Wt1Cre*-derived cardiomyocytes likely derive from early extra myocardial Wt1Cre-expressing cells and not from established epicardium

**Figure 4:**
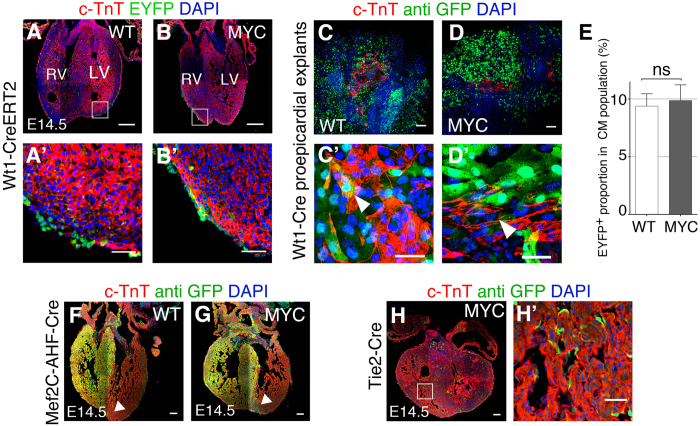
The *Wt1Cre*-recombined cardiomyocytes do not derive from the epicardium. (**A,B**) Confocal images of histological sections from E14.5 hearts *iMOS*^*WT*^(WT) (**A**) and *iMOS*^*T1-Myc*^(MYC) (**B**); *Wt1-Cre ERT2*. **A’,B’** show a magnification of the boxed areas shown in (**A,B**). (**C**–**D’**) Proepicardial explants cultured for 48 h from *iMOS*^*WT*^(WT) (**C**) and *iMOS*^*T1-Myc*^(MYC) (**D**) where big clusters of beating cells were detected to be positive for cadiac TnT staining (arrowheads). **E** Quantification of the proportion of cardiomyocytes that expressed EYFP. (**F**,**G)** Confocal images from histological sections of *Mef2C-AHF-Cre-*recombined *iMOS*^*WT*^(WT) (**F**) and *iMOS*^*T1-Myc*^(MYC) (**G**) mosaic hearts at E14.5. Arrowheads show the front of EYFP cardiomyocyte colonization. (**H,H’**) Confocal images from histological sections of *iMOS*^*T1-Myc*^(MYC) E14.5 heart induced with *Tie2-Cre*. **H’** shows a magnification from the boxed area in (**H**). Graph in **E** shows means +SEM. *p < 0.05; **p < 0.01; ***p < 0.001. Bar, 100 μm in **A,B,C,D,F,G,H** and 20 μm in **A’,B’,C’,D’,H’**, LA left atrium, LV left ventricle.

To investigate the origin of EYFP cardiomyocytes in the *Wt1Cre*-recombined *iMOS*^*T1-Myc*^ hearts, we induced recombination using a tamoxifen (TM)-inducible Cre driver (*WT1CreERT2*). This line has been reported before to induce limited recombination of the epicardium and to contribute to the cardiomyocyte lineage in one study^15^ but not in other^12^. Here we optimized the tamoxifen administration protocol to obtain around 50% recombination of the E14.5 epicardium. We administered TM at E9.5, which produced epicardial *iMOS* recombination at E10–10.5 (not shown), however contribution of *iMOS*^*+*^ cells to cardiomyocytes at E14.5 was neither observed in the control, nor in the Myc-expressing mosaics, even though EPDCs were detected (Fig. 4A,B’). These results suggested that *Wt1Cre*-derived cardiomyocytes do not derive from the epicardium and are in agreement with the absence of cardiomyocyte differentiation from *Wt1Cre*-recombined *iMOS*^*T1-Myc*^ epicardium in plain explants and in the co-culture assays (Fig. 3D,E’,G–J).

To further study this aspect, we analyzed pro-epicardial explants, which have been shown to partially differentiate to the cardiomyocyte lineage (Fig. 4C,D’). Although we observed differentiation to cardiomyocytes in derivatives of the explanted proepicardia, EYFP-Myc and EYFP-wild type cells contributed equally to this lineage (Fig. 4C,D’), indicating that Myc overexpression does not enhance differentiation to cardiomyocytes in this assay (Fig. 4E).

We then investigated other possible origins of the *Wt1Cre*-labeled cardiomyocytes. Since cardiomyocytes are observed in the IVS of the *Wt1Cre*-recombined *iMOS*^*WT*^ hearts, we considered the possibility that cell competition could stimulate the colonization of the ventricular free walls by expanding the original septal cardiomyocyte population. To explore this possibility, we recombined *iMOS*^*WT*^ and *iMOS*^*T1-Myc*^ hearts with the *AHFCre* line, which produces recombination in the RV and IVS. Although we could observe local expansion of the EYFP-Myc population in E14.5 hearts, we did not detect colonization of the LV free wall as previously detected when using the *Wt1Cre* line (Fig. 4F,G), which discards septal cardiomyocytes as the origin of the LV free wall contribution of the *Wt1Cre* lineage.

We observed that the *Wt1Cre* line also recombined some hematopoietic cells and endocardial cells from early stages of cardiac development (Fig. 4H,H’). To explore whether these lineages could generate the *Wt1Cre*-recombined *iMOS*^*T1-Myc*^ cardiomyocytes, we used the *Tie2Cre* line, which recombines all endothelial and blood lineages in the mouse embryo. We did not observe any contribution of the *Tie2Cre* lineage to the cardiomyocytes in E14.5 *iMOS*^*T1-Myc*^ hearts (Fig. 4H,H’), which discarded blood or endothelial precursors as the origin of *Wt1Cre*-derived cardiomyocytes.

**Figure 5:**
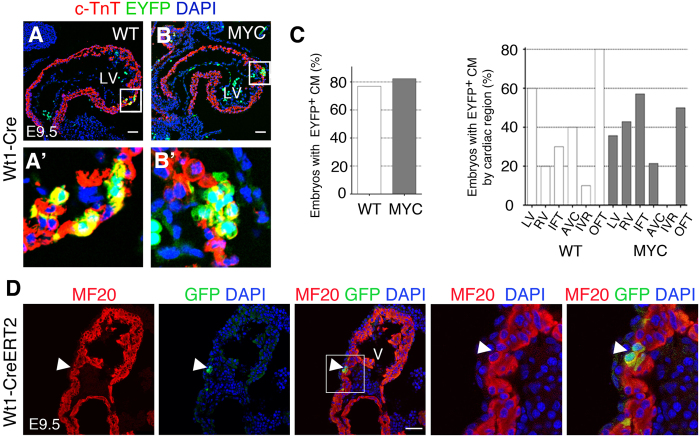
Myc overexpression does not affect the initial pattern of recombined cardiomyocytes (**A,B**) Confocal images of histological sections from E9.5 hearts *iMOS*^*WT*^(WT) (**A**) and *iMOS*^*T1-Myc*^(MYC) (**B**) stained for c-TnT. **A’** and **B’** show magnification of boxed areas in (**A**–**C)**, Graphs showing the proportion of embryos showing Wt1 lineage contribution to cardiomyocytes in *iMOS*^*WT*^(WT) and *iMOS*^*T1-Myc*^at stage E9.5 (left) and the distribution of Wt1 contribution to different areas of the heart in *iMOS*^*WT*^(WT) and *iMOS*^*T1-Myc*^(MYC) at stage E9.5 (right). (**D)**. Detection of Cre-recombined cardiomyocytes in the E9.5 heart following recombination induction at E8.0 in the *Wt1-Cre ERT2* line. From left to right, images show MF20 expression, GFP detection and their co-localization. Panels to the right show a magnification of the boxed area. Bar is 50 μm. V, ventricle of the early cardiac tube. Graphs in (**C**) show means ± SEM. n ≥ 3 hearts in (**A–C**), n ≥ 5 in D.

We then studied the timing and site of appearance of *Wt1Cre*-recombined cardiomyocytes in *iMOS*^*WT*^ and *iMOS*^*T1-Myc*^ hearts. We did not detect any *iMOS*^*+*^ cardiomyocytes before E9 (data not shown, N = 5), which discards recombination in early cardiac mesodermal precursors. The first EYFP cardiomyocytes were detected in both *iMOS*^*WT*^ and *iMOS*^*T1-Myc*^ hearts between E9 and E9.5 before colonization by epicardial cells (Fig. 5A,B’). At this stage, the frequency of hearts containing EYFP cardiomyocytes was similar in *iMOS*^*WT*^ and *iMOS*^*T1-Myc*^ hearts and the localization of EYFP cardiomyocytes in different regions of the developing heart is very variable but similar in control and Myc mosaics (Fig. 5C). These results show that recombination of the cardiomyocyte lineage is induced by *Wt1Cre* in a scattered and non-stereotyped manner in both *iMOS*^*WT*^ and *iMOS*^*T1-Myc*^ hearts and that Myc overexpression does not affect the initial pattern of recombined cardiomyocytes. In addition, these results show that the recombined cardiomyocytes should derive from *WT1Cre*-expressing cells before the establishment of the epicardium. To confirm this possibility, we induced Cre activity in the *WT1CreERT2* line by TM injection at E8.0 and observed scattered recombined cardiomyocytes at E9.5 (Fig. 5D), contrary to what was observed following induction at later stages, in which no recombined cardiomyocytes were detected (Fig. 4A,B’).

**Figure 6:**
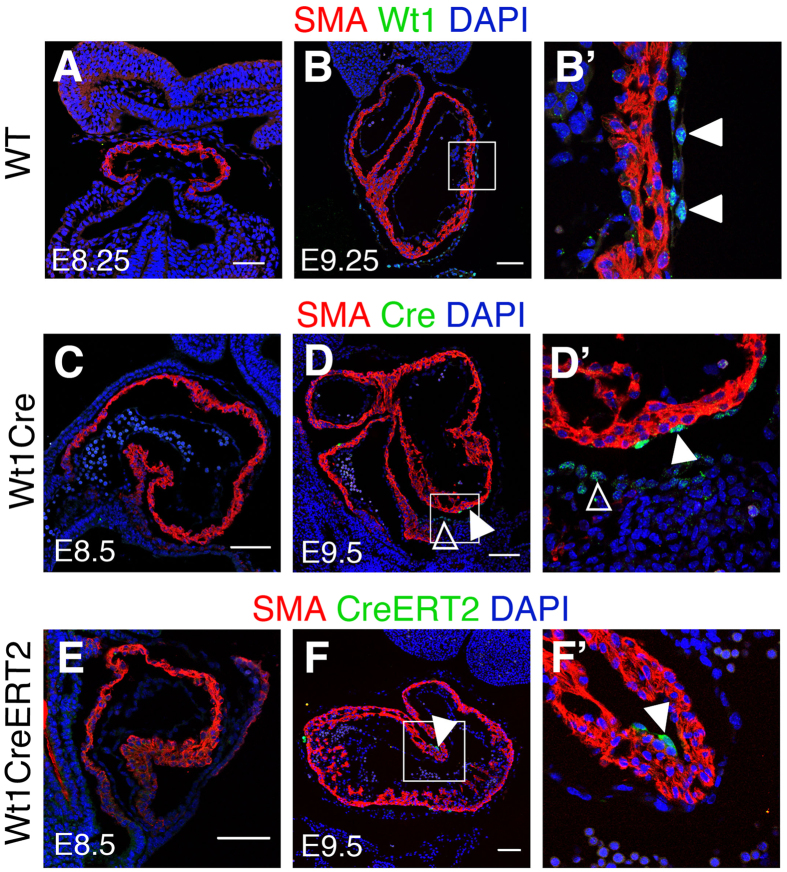
Cardiomyocyte recombination does not correlate with Cre or CreERT detection in cardiomyocytes (**A**,**B)** show immunofluorescence with anti-Wt1 and anti-SMA antibodies on sections of the developing cardiac tube of wild type embryos. (**B’**) shows a high magnification from the boxed area in (**B**). Arrowheads indicate pericardial Wt1^+^ cells. (**C**,**D**) show immunofluorescence with anti-Cre and anti-SMA antibodies on sections of the developing heart tube of *Wt1Cre* embryos. (**D**’) shows specific areas magnified from (**D**). Solid arrowheads indicate Cre-positive epicardial cells; empty arrowheads indicate Cre-positive pro-epicardial cells. (**E**,**F**) show immunofluorescence with anti-ER and anti-SMA antibodies on sections of the developing heart tube of *Wt1CreERT2* embryos. (**F**’) shows specific areas magnified from **F**. Solid arrowheads indicate CreERT2-positive epicardial cells. Bar is 50 μm in (**A**,**B**) and 100 μm in (**C–F)**.

We then tried to identify the original cells in which the recombination was produced and specifically whether cardiomyocytes undergo Cre recombination *in situ*. The fact that two different Cre lines driven by *Wt1* regulatory sequences led to early cardiomyocyte recombination suggested that this activity could correspond to endogenous *Wt1* expression. To explore this possibility, we studied in detail Wt1 protein expression in early cardiac formation, with a special emphasis on the stages in which *Wt1Cre* recombination was first observed (Fig. 6). The first Wt1^+^ cells were observed around E9 in the pericardial and proepicardial regions (Fig. 6B,B’ and data not shown), however, we did not detect any Wt1-positive cardiomyocyte between E8 and E14.5 (Fig. 6A,B’ and data not shown). To determine whether, independently of *Wt1* expression, the *Wt1Cre* and *Wt1CreERT2* lines used here produced Cre protein expression directly in cardiomyocytes, we performed antibody staining of Cre and CreERT2 proteins (Fig. 6C–F’). While Cre and CreERT2 could be clearly detected in the epicardium and pro-epicardium, both proteins were completely absent from cardiomyocytes between E8 and E10 (Fig. 6C–F’). Altogether our results suggest that the observed recombined cardiomyocytes derive from extra-myocardial *WT1Cre*-expressing cells before the establishment of the epicardium.

## Discussion

We reported here the ability of Myc moderate overexpression to provoke homeostatic cell population shifts during the development of the epicardial lineage. Myc-induced preferential expansion took place from the first stages of epicardial development, being observable in primary cultures of E10.5-explanted epicardial cells, and further increased at various stages of epicardium and epicardial-derived cells development, including the myocardium invasion and the colonization of the vasculature. The tissues colonized by EPDCs have multiple origins, with EPDCs contributing to only a fraction of these tissues. Interestingly, in most cases we observed that the expansion of EYFP-MYC cells occurred within the EPDC compartment of the colonized tissues, without displacing cells from other sources than EPDCs. Exceptions to these observations were the microvasculature endothelium and the arterial smooth muscle, where we observed clear overexpansion of the EPDC compartment with displacement of cells from other origins. Interestingly arterial coronary smooth muscle, but not venous coronary smooth muscle receives a contribution from the neural crest^31^, suggesting MYC-stimulated EPDCs may be able to displace neural crest smooth muscle precursors but not those of different origins. Similarly, large part of the arterial coronary endothelium is produced by budding of well-established endothelial sacs from the endocardium^32^, while the microvasculature is produced by other vasculogenic/angiogenic mechanisms. These differences in the mechanisms for the generation of endothelial populations may then underlie the different colonizing activity of Myc-stimulated EPDCs.

In part, the expansion of EYFP-MYC cells could take place by cell competition, however Myc-induced cell competition in mouse development involves close interactions between cells that are expected to take place in established tissues, like fully-colonized epicardium or vascular layers. In the case of epicardium-EPDC development, a very important additional step could be involved in the enrichment; cell migratory and invasive ability. In fact, we observed that Myc cells showed enhanced migratory and myocardium-invasive ability in explant co-culture assays and the complete colonization of the vasculature by Myc-overexpressing cells may involve an enhanced migratory ability. These observations suggest that stimulating epicardial cell activity through homeostatic Myc overexpression may enhance the potential reparative ability of epicardium and epicardial-derived cells.

In addition, we observed widespread colonization of the ventricular free walls by *Wt1Cre*-derived cardiomyocytes in Myc mosaics but not in control mosaics. However, a detailed study in cultured explants failed to show any ability of Myc to stimulate cardiomyocyte differentiation. Furthermore, *Wt1CreERT2*-recombined epicardial cells failed to contribute to the cardiomyocyte lineage, even when Myc was overexpressed. Given the ability of Myc to expand cardiomyocyte populations in the developing myocardium, even very minor initial contributions from the epicardial lineage to cardiomyocytes would have been expanded and easily detected. Our results thus strongly support the inability of Wt1^+^ epicardial cells to contribute to the cardiomyocyte lineage during development^12^ consistent with observations in other vertebrate models^7^,^33^. Based on our Cre and CreERT2 expression analysis, we suggest that Cre activity in *Wt1Cre*-expressing precursors before epicardium adhesion to the ventricles could be the origin of cardiomyocyte lineage colonization, however we cannot completely exclude *in situ* cardiomyocyte recombination driven by undetectable levels of Cre protein expressed in cardiomyocytes. Our study of the *Wt1Cre* and *Wt1CreERT2* lines thus suggests the exclusive use of the latter when aiming for targeting Wt1^+^ epicardial-derived lineages, with the caveat that also endocardial/endothelial cells express *Wt1* and undergo Cre-recombination in the *WT1CreERT2* line^12^. Surprisingly, the initial pattern of *Wt1Cre* recombination in cardiomyocytes is similar in control and Myc mosaics, however, at E14.5, cardiomyocytes in the free ventricular walls are only detectable in the Myc mosaics, while labeled cardiomyocytes in the IVS are observed in both. This pattern of colonization, and the *in vitro* studies reported here, indicate that Myc does not regulate the incorporation of Wt1Cre lineages to the cardiomyocyte pool, but differentially expands this initial population. Interestingly, the epicardium is essential for proper IVS formation and has an important cellular contribution to the IVS in different species^34^, however contribution to the IVS cardiomyocytes has not been observed in Quail-Chick transplantation experiments^34^.

The overcolonization of the ventricles by EYFP-MYC cardiomyocytes in Myc mosaics continues progressing until birth with up to 50% colonization (in some specimens) of the left ventricle free wall cardiomyocytes, while it remains barely detectable in control mosaics. Interestingly, the initial recombination pattern seems to affect more the derivatives of the first heart field (FHF) (See Fig. 1A), however at later stages, colonization of the RV progresses to reach the levels observed in the LV. Very likely this effect is related to the observation that *Wt1Cre*-promoted cardiomyocyte recombination takes place mostly during early cardiac stages, when only FHF cardiomyocytes are in place. In fact, using the *AHFCre* line, we observed expansion of second heart field (SHF) derivatives into the FHF-derived myocardium (Fig. 4G), indicating that Myc enhancement confers cardiomyocytes the ability to violate the boundary between FHF and SHF.

Myc-driven phenotypically silent cell competition in cardiomyocytes has been previously reported, however the results reported here highlight the extraordinary ability of cell competition to stimulate small cardiomyocyte populations to colonize extensive areas of the myocardium. Our results suggest that cell competition stimulation could be useful for enhancing epicardial activation and cellular contributions to the myocardium. In addition, they highlight an extraordinary capacity of cell competition to expand scarce cardiomyocyte populations without altering cardiac anatomy.

## Methods

### Mouse Strains

All experiments were performed using mice (Mus musculus) of a mixed background that were maintained and handled according to the recommendations of the CNIC Ethics Committee, the Spanish laws and the EU Directive 2010/63/EU for the use of animals in research. The experimental protocols involving animals were approved by the CNIC and Universidad Autónoma de Madrid Committees for “Ética y Bienestar Animal” and the area of “Protección Animal” of the Comunidad de Madrid with reference PROEX 220/15.

*iMOS* Mouse lines have been previously described 28. Homozygous *iMOS* females were mated with males carrying different Cre lines.; *Wt1Cre*^30^, *Mef2C-AHFCre*^35^, *Tie2Cre*^36^ and *Wt1Cre ERT22*^10^ to generate embryos or pups. Mice were genotyped by PCR. To induce recombination in *iMOS;Wt1CreERT2* mice, tamoxifen was administered by oral gavage (4 mg/mL). The Wt1GFP knockin line^37^, in which the exon 1 of one Wt1 allele has been replaced by the GFP sequence, was also used as an independent, reporter for active Wt1 transcription.

### Embryonic epicardial explants

We followed previously described methods^38^. Briefly, to isolate epicardial cells from E10.5 and E11.5 embryos, hearts were dissected and the ventricles were each cut into two pieces. Each piece was placed with the epicardial outermost part facing down onto a gelatin covered MatTek Glass bottom Dish (0.1%Gelatin in PBS). These myocardial pieces were cultured in DMEM containing 10% FBS and 1% Penicillium Streptomycin. After 24 to 48 hours, epicardial cells had migrated from the explant and form a monolayer and the myocardial explant was removed using forceps. Epicardial cells were left to grow for 5 days, at which point they were visualized by confocal microscopy and/or fixed for immunostaining in 2%PFA overnight at 4 °C. Coexplant assays were performed in a similar way but adding a E9.0 heart in the dish close to the epicardial explant without contacting it.

### Embryonic proepicardial explants

This method is extensively described in^39^. Embryos for proepicardium explants were harvested at E9.0. The proepicardium was identified just under the heart as a “grape-like” clustering of cells. Heart tube was removed and discarded and proepicardial cells were removed. Proepicardial explants were cultured in DMEM 10% FBS 1% Penicillin-Streptomycin for 48 h on gelatin coated MatTek Glass bottom dishes.

### Confocal microscopy

Histological sections and epicardial explants were imaged with a Nikon A1R confocal microscope using 405, 458, 488, 568 and 633 nm wavelenghts and 20x/0.75 dry and 40/1.30 oil objectives. Areas occupied by EYFP and ECFP cells and EYFP and ECFP cell number were quantified using the threshold detection and particle analysis tools of the Image J, (NHI, http://rsb.info.nih.gov/ij). To calculate the relative frequency of ECFP cells the percentage of ECFP cells in each embryo was divided by the average percentage in *iMOS*^*WT*^ mosaic. ECFP was scored by substracting the information of EYFP from the anti-GFP staining which detects both fluorescent proteins.

### Immunofluorescence

Embryos were obtained at different gestational stages, fixed overnight at 4 °C in 2% paraformaldehyde in PBS, gelatin embedded and cryosectioned. Embryonic epicardial and proepicardial explants were fixed in 2% PFA overnight at 4 °C and whole stained. Primary antibodies used were Wt1 (ab89901 abcam), Living colors Rabbit polyclonal anti GFP antibody (Clontech), c-TnT (MS-295 Thermo Scientific), PECAM-1 (553370 BD Pharmingen™ clone MEC 13.3), SM22a (ab14106 abcam), SMA (C6198 Sigma), MF20 (DSHB), PH3 (06-570 Millipore), Cre (69050 Millipore) and ER (ab27595 abcam). For coronary vessel characterization, intima was detected with PECAM-1, media with SM22a and adventitia cells were identified by their mesenchymal appearance and their disposition surrounding the media. Veins and arteries were identified by their position within the ventricle (veins more superficial and arteries more internal) and by the thickness and shape of the cells of the media, which are thin and fusiform in veins and thick and cuboidal in arteries. Identification with specific antibodies was not possible due to the need to detect 4 different channels in our experiments. In experiments in which both ECFP^+^ and EYFP^+^ positive cells were characterized, we found that ECFP was difficult to detect after section processing. We therefore detected all FPs with anti-GFP immunofluorescence and EYFP through its native fluorescence. Cells positive for anti-GFP and negative for EYFP were considered ECFP^+^.

### Flow cytometer

Isolated cardiac cells from embryonic hearts were analyzed by flow cytometry to quantify EYFP population (ECFP was undetectable by flow cytometry). Propidium Iodide was added to the cells to asses viability (1:5000). An LSR Fortessa 4L Flow Cytometer was used for the analysis (Laser wavelengths 488, 640, 405, 561). For the analysis, FACSDiva and FlowJo softwares were used.

### Statistical analysis

To compare average percentages of ECFP cells between more than two groups, the Kruskal-Wallis test was used (assuming non-normal distributions). For comparisons of two groups a Man-Whitney U-test was used. All comparisons were made using Prism statistical software.

## Additional Information

**How to cite this article**: Villa del Campo, C. *et al*. Myc overexpression enhances epicardial contribution to the developing heart and promotes extensive expansion of the cardiomyocyte population. *Sci. Rep.* **6**, 35366; doi: 10.1038/srep35366 (2016).
