# Erratum: Myc overexpression enhances epicardial contribution to the developing heart and promotes extensive expansion of the cardiomyocyte population

**DOI:** 10.1038/srep37880

**Published:** 2016-12-09

**Authors:** Cristina Villa del Campo, Ghislaine Lioux, Rita Carmona, Rocío Sierra, Ramón Muñoz-Chápuli, Cristina Clavería, Miguel Torres

Scientific Reports 6: Article number: 3536610.1038/srep35366; published online: 10 18 2016; updated: 12 09 2016

The original version of this Article contained a typographical error in the title, where:

“Myc overexpression enhances of epicardial contribution to the developing heart and promotes extensive expansion of the cardiomyocyte population”.

Now reads:

“Myc overexpression enhances epicardial contribution to the developing heart and promotes extensive expansion of the cardiomyocyte population”.

This has now been corrected in the HTML and PDF versions of this Article.

